# Rethinking the Complexation of Phytochemicals in Natural β-Cyclodextrins

**DOI:** 10.34172/apb.43888

**Published:** 2024-10-30

**Authors:** Ian Jhemes Oliveira Sousa, Rita de Cássia Meneses Oliveira

**Affiliations:** ^1^Northeast Biotechnology Network, Focal Point on - Federal University of Piauí – UFPI.; ^2^Department of Biophysics and Physiology, Federal University of Piauí – UFPI.

## To Editor,

 β-Cyclodextrins (β-CD) have been widely used in the complexation of bioactive compounds for decades, especially those with low aqueous solubility.^1-3^ However, there is a limitation little discussed in the literature when it comes to the efficiency of this complexation, particularly with insoluble substances of a liquid nature, such as terpenes, phenylpropanoids and other secondary metabolites.

 The rigid structure and low aqueous solubility of natural β-CD, not discussed, is a factor that raises many important points of observation of merit, since a large number of authors still base their possibility of increasing solubility and or even improving bioavailability on proposals for complexation with β-CD. In aqueous media, the solubility of β-CD results in limited efficiency in the formation of stable complexes that clump together and form aggregates in aqueous media, which limits its own solubility^4^

 This aspect, although chemically recognized, has not been widely discussed, leading to an indiscriminate use of natural cyclodextrins in attempts to improve the bioavailability of hydrophobic compounds, especially natural products.

 The low complexation efficiency of β-CDs with liquid substances has not been explored as it should be, which leads to a large contingent of research costs resulting in complexes with low yield and low complexation effectiveness.

 In this context, the phase solubility diagram model proposed by Higuchi and Connors^5^ seems to have been somewhat forgotten in numerous plans for the development of inclusion complexes, since they continue to explore the possibilities of natural β-CDs even in the face of results of low complexation efficiency and charge.

 This letter aims to “rekindle” the discussion about the appropriate selection of the cyclodextrin host for different types of bioactive substances.

 The use of the Higuchi and Connors model can offer important insights to observe the limitations of natural cyclodextrins and the possibilities of modified cyclodextrins, such as hydroxypropyl-β-cyclodextrin, methyl-β-cyclodextrin or even dimethyl-β-cyclodextrin, in order to obtain an inclusion complex with better values ​​of complexation efficiency and transport efficiency in relation to an isosmolar ratio of the complexing agent and the complexation candidate.

 This observation comes to light as a commitment from a researcher who was frustrated when trying to perform complexations of a phenylpropanoid isolated from the essential oil of a plant whose complexation efficiencies were often low, regardless of the technique used, due to the characteristics of natural β-CD.

 Therefore, I am bringing this discussion to the surface so that the scientific community may redouble its attention to the limitations of natural cyclodextrins and explore in greater depth the advantages of modified cyclodextrins, especially in studies involving compounds with low solubility and liquid characteristics, such as those derived from essential oils.

 The adoption of modified cyclodextrins in formulations could not only increase therapeutic efficiency, but also open new avenues for the application of hydrophobic bioactive compounds.

## Competing Interests

 None declared.

## Ethical Approval

 Not applicable.
